# Memory for faces and voices varies as a function of sex and expressed emotion

**DOI:** 10.1371/journal.pone.0178423

**Published:** 2017-06-01

**Authors:** Diana S. Cortes, Petri Laukka, Christina Lindahl, Håkan Fischer

**Affiliations:** Department of Psychology, Stockholm University, Stockholm, Sweden; University of Würzburg, GERMANY

## Abstract

We investigated how memory for faces and voices (presented separately and in combination) varies as a function of sex and emotional expression (anger, disgust, fear, happiness, sadness, and neutral). At encoding, participants judged the expressed emotion of items in forced-choice tasks, followed by incidental Remember/Know recognition tasks. Results from 600 participants showed that accuracy (hits minus false alarms) was consistently higher for neutral compared to emotional items, whereas accuracy for specific emotions varied across the presentation modalities (i.e., faces, voices, and face-voice combinations). For the subjective sense of recollection (“remember” hits), neutral items received the highest hit rates only for faces, whereas for voices and face-voice combinations anger and fear expressions instead received the highest recollection rates. We also observed better accuracy for items by female expressers, and own-sex bias where female participants displayed memory advantage for female faces and face-voice combinations. Results further suggest that own-sex bias can be explained by recollection, rather than familiarity, rates. Overall, results show that memory for faces and voices may be influenced by the expressions that they carry, as well as by the sex of both items and participants. Emotion expressions may also enhance the subjective sense of recollection without enhancing memory accuracy.

## Introduction

Recognizing and remembering specific people is a central aspect of daily interactions. These processes are often influenced by emotional and social nonverbal expressions that people infer from others’ facial appearance and tone of voice [1]. Sex also plays a key role in social remembrance, as research suggests that women perform better than men in face recognition tasks. In particular, several studies have reported evidence for a female own-sex bias in memory to the effect that women remember more female than male neutral faces (for a review, see [2]). We note that the previous studies on memory bias have focused heavily on the facial channel, leaving aside other important person characteristics such as the voice. In addition, previous studies have mainly included neutral stimuli (e.g., [2, 3, 4]), despite the importance of emotional expressions for human interactions. The current study therefore aims to investigate effects of item sex and participant sex, as well as the effect of emotion expression, on memory for both faces and voices.

### Own-sex bias in memory for faces and voices

Results showing female own-sex bias for neutral faces are fairly consistent across studies (e.g., [2]), but results for men show more variability. Some studies have reported that men remember neutral faces of both sexes at similar levels [5, 6], whereas others have reported that men, just like women, are more accurate at recognizing female compared to male faces [4, 7]. There are even some reports of male own-sex memory bias, where male participants showed superior performance for male neutral faces [8, 9].

It has been suggested that men and women may use different strategies during the encoding phase [2, 7, 10, 11], with women focusing more than men on emotional expressions [12]. Fulton et al. [12] instructed participants to specifically attend to the expression of faces (happy or neutral) and demonstrated that men’s recognition of own sex faces improved, and was similar to women’s recognition of male faces, when directly allocating attention to the expression rather than the sex of the face. There is also evidence for neural correlates of own-sex bias in memory. For example, women showed higher activation in areas relevant to face processing during encoding for female faces compared to male faces while this pattern was not found for male participants [13].

Few previous studies have investigated own-sex bias for emotionally expressive faces, and it is currently unclear if and how own-sex bias varies as a function of emotion. Wang [11] investigated if there was a female memory advantage for neutral, angry and happy expressions. The results indicated that while women outperformed men in overall recognition accuracy for female faces, this effect was driven by happy faces. In contrast, Armony and Sergerie [14] reported an own-sex memory bias for female participants that was limited to fearful faces when presenting neutral, happy and fear expressions. Wang [15], however, failed to replicate findings of own-sex bias in a study including neutral, positive, fearful, angry, sad, surprised, and disgusted faces.

Emotions are not only expressed through facial behavior, vocal expressions are also prime sources of information about a person’s affective state, both separately and in combination with facial expressions. As far as we know there are no previous studies on sex bias in memory for emotional voices. However, in a related study Skuk and Schweinberger [16] investigated sex differences in identification of familiar emotionally neutral voices, and reported that men identified more male than female voices, whereas women were equally good at identifying voices from both sexes, and showed higher overall identification rates than men.

### Memory for emotional faces and voices

Previous research is generally inconclusive regarding the effect of emotional expressions on unfamiliar face identity memory. Several studies report that memory for positive faces is more accurate than for neutral or negative ones [17, 18, 19, 20]. However, other studies have instead found the opposite effect with an advantage for negative faces [14, 21, 22], while yet some studies report no clear advantage for emotional compared to neutral faces [23]. We note that it is difficult to directly compare results across studies because they differ in their methodology (e.g., incidental versus intentional memory tasks), and aim to address slightly different research questions. While in some studies no identities were repeated during encoding phase [14, 15, 20], other studies showed each identity several times but displaying different emotions [17, 18, 20]. In addition, different stimuli from the same identities have been used at study and test phases, for example, emotional faces were presented only in the encoding session whereas neutral faces were shown in the test session [17, 19, 20, 21].

Importantly, studies vary regarding which emotions they have included, with most of them including only few emotion categories. In one of the most comprehensive studies in this respect, Liu et al. [19] compared the effects of anger, disgust, fear, happiness, sadness, and surprise expressions on facial identity recognition by changing expressions in the retrieval phase. When the same expression was shown at both encoding and recognition, memory for happy expressions was better than for disgusted expressions, but not significantly different from the other expressions. Although they included a wider selection of emotions than prior studies, Liu et al. [19] did not examine recognition of neutral expressions, making it hard to draw conclusions about whether emotional faces show an advantage compared to non-emotional faces. We argue that it is essential that studies on recognition memory include several emotion categories as well as neutral expressions in order to elucidate which emotion expressions, if any, enhance accuracy.

There are only a couple of studies on memory for vocal emotion expressions. Armony, Chochol, Fecteau, and Belin [24] as well as Aubé, Peretz, and Armony [25] reported that unfamiliar emotional non-linguistic vocalizations (e.g., screams, laugher, cries) conveying fear, happiness, and sadness were better remembered than neutral vocalizations. Memory for voices thus seems to depend on the emotions that are being expressed, but just as for faces, the effect of individual emotions remains largely an open question.

### Recollection and familiarity processes

Studies have recently started to investigate the states of awareness associated with recognition of faces and voices. By using the Remember/Know procedure [26], it is possible to study if own-sex bias and memory for emotional expressions are influenced by recollection or familiarity processes. Remember responses are accompanied by recollection of details or specific contextual information (e.g., thoughts, feelings, and associations) about the stimuli that have been presented and are therefore related to episodic memory. In contrast, know responses are associated with feelings of familiarity about the stimuli, in the absence of retrieval of contextual information about the prior experience [26].

Findings indicate that emotions may affect remember and know judgments in different ways. More specifically, it has been suggested that emotions may facilitate the subjective sense of recollection (e.g. [27, 28, 29]). For example, Patel, Girard, and Green [30] reported that memory for angry, happy and fearful unfamiliar faces may depend more on recollection processes. In contrast, Johansson et al. [23] found that memory for happy and neutral faces instead may depend on familiarity while negative stimuli rely on recollection processes. The mechanisms behind why certain emotions are associated with recollection rather than familiarity processes still need to be addressed. Fearful and angry expressions are considered as highly arousing which may facilitate recollection processes [23, 27], and although happy expressions can be considered as high in arousal too, they are probably not as relevant (or as aroused) as fearful or angry expressions and therefore classified as know responses. When participants are instructed to explicitly attend and process the displayed emotion, recollection rates for happiness improve [30]. Thus, recollection and familiarity processes may be influenced by both the emotional expression of the stimuli and the encoding strategy that is used.

Damjanovic and Hanley [31] assessed to what extent recognition of emotionally neutral famous faces and voices was accompanied by either recollection of specific episodes or a sense of familiarity without conscious recollection. It was easier to recall episodic information from faces than from voices, and voices were more associated with familiarity ratings compared to faces. These results held true even when participants were exposed to personally familiar faces and voices [32]. A possible explanation is that faces, which evoked more remember responses, may be related to episodic memory; whereas voices provide less information since no details or contextual information were recalled during their presentation [31, 32, 33]. Damjanovic and Hanley [31] therefore proposed that episodic memory is more robustly linked to face recognition than to voice recognition.

### Study rationale

Daily interactions are characterized by meeting people of different sexes who often express different emotions through their facial and vocal behaviors, and we argue that this implies that research on social memory bias should expand beyond the use of neutral faces as objects of study. The current study therefore includes male and female items and participants, three presentation modalities (faces only, voices only, and face-voice combinations), and stimuli expressing a fairly large number of expressions (anger, disgust, fear, happiness, and sadness) as well as neutral items. This design allows us to build upon the literature reviewed above by investigating the effects of sex and emotion expression on memory for both faces and voices.

We aim to address three interrelated research questions. First, we investigate if own-sex bias in memory accuracy is exclusive for faces (and women) or if it can also be observed for voices and face-voice combinations (and men). Second, we investigate the effects of emotion expression on memory accuracy (and own-sex bias). Here, we are especially interested in which emotion expressions, if any, enhance or decrease memory accuracy, and if the patterns of results for specific emotions are similar for faces, voices, and face-voice combinations. Third, we use the Remember/Know paradigm to investigate if the subjective sense of recollection and familiarity varies as a function of sex and expression, and if they show evidence for own-sex bias. Based on previous studies (e.g., [2, 4]), we expect to replicate findings of female own-sex bias for faces. For the other research questions, our analyses provide novel data on issues that have not been previously addressed or for which previous studies provide inconclusive results.

## Methods

### Participants

A total of 600 young adults were recruited from the Stockholm area through university bulletin boards and designated websites. All participants were right-handed, fluent in Swedish, and reported being in good health with no previous or current substance abuse or psychiatric medication. One participant withdrew from the study, and data from 3 participants was lost due to equipment failure, leading to a total of 596 participants (226 men, mean age = 23.4 years, *SD* = 3.16, range 18–34; 367 women, mean age = 22.9, *SD* = 3.16, range = 18–34). Data on age and sex was not available for 3 participants; data from these individuals were included in all analyses, except those involving sex as a variable of interest. Participants received movie vouchers or course credits in exchange for their participation. Written consent was obtained from all participants prior to data collection, and the study was approved by the Stockholm Area Regional Ethical Review Board. Data was collected in the framework of a larger project investigating genetic and neural correlates of emotional abilities, and the sample size was determined a-priori based on considerations of expected strength of phenotype-genotype correlations.

### Materials

#### Face and voice stimuli

Face stimuli were retrieved from the FACES database [34], which contains color photos of young and middle-aged adults who portray various emotions. All photos were portraits of faces (335 x 419 pixels) including hair, with head and gaze oriented forward, and all expressers wore similar clothes. The voice stimuli were taken from the VENEC corpus [35], which contains recordings of young adult actors expressing various emotions by way of non-linguistic vocalizations. The vocalizations consisted of various human sounds (e.g., crying, laughter, shrieks) and non-linguistic interjections (e.g., “ah”, “hm”, “oh”), but contained no actual words. The amplitude of the voice stimuli was peak normalized using *Adobe Audition* software (Adobe Systems Inc., San Jose, CA, USA) to control for differences in recording level. The duration of voice stimuli varied between 1 to 4 seconds.

Twenty-four stimuli expressing anger, disgust, fear, happiness, neutrality and sadness were shown at encoding (4 stimuli/expression) for each presentation modality (faces only, voices only, and face-voice combinations). During the recognition memory task, the 24 previously encountered stimuli were interspersed with 24 new stimuli–resulting in a total of 48 stimuli per presentation modality. The selection criteria for both face and voice stimuli were (a) that the intended emotion of the item was recognized with high accuracy in previous studies [34, 35], (b) that the item pool would include equal numbers of female and male expressers for each emotion and presentation modality, and (c) that each expresser identity would occur only once for each presentation modality. Face stimuli further contained equal proportions of young and middle-aged expressers for each condition. For the face-voice combinations, the face and the voice stimuli were presented simultaneously, and were matched in terms of expression and sex. The names of all stimulus items are available in the online supplemental S1 Table (for replication purposes).

### Procedure

Testing was conducted individually using *MediaLab* software (Jarvis, 2010) to present stimuli and collect responses. Face stimuli were presented on 22-inch LED computer screens and participants listened to the voice stimuli at comfortable volume levels through high-quality headphones, with sound level kept constant across participants. All participants first took part in the encoding and recognition memory tasks (described below) and then participated in additional tasks (an emotion recognition task and a questionnaire battery focusing on socio-emotional abilities and functioning) not related to the current study. In addition, all participants provided saliva samples for genetic analyses. Results on the genetic correlates of social memory are reported elsewhere [36, 37].

#### Encoding task

Participants were not told that they would undergo a memory task because we were interested in incidental learning. Instead they were instructed to judge the expression of faces, voices, and face-voice combinations in a forced-choice task, by clicking (using their dominant hand) on the label which best represented the expression conveyed by each stimulus. The alternatives they could choose among were the same as the intended expressions (anger, disgust, fear, happiness, neutral, and sadness), and a response was scored as correct if it matched the intended expression of the stimulus. Participants did not receive any feedback regarding their responses while performing the expression identification task. After the participants had finished the face encoding task, they similarly judged the expression of the voice stimuli which were then followed by the face-voice combinations. The order of stimuli was randomized within each encoding task (but all participants saw/heard exactly the same stimuli). The order of encoding tasks (i.e., faces, voices, and face-voice combinations) was, however, not randomized because we were not interested in comparing modalities in the current study. In this way, we could also keep possible order effects constant for the genetic association studies mentioned above [36, 37].

Participants were instructed to make their judgments as quickly and accurately as possible, but there was no time limit on the judgments (the next stimulus appeared immediately after the participants made their judgments). Stimuli in the face only modality remained on the screen until the participants made their judgments, which usually took approximately 3–5 seconds. Participants were allowed to repeat the playback of voice stimuli as many times as needed to reach a decision, to compensate for the brief duration (1–4 seconds) of the vocalizations. However, in the face-voice combinations, the vocalizations were only played once and could not be repeated. Here, the faces and the voices had the same onset (they appeared simultaneously), but the offset differed because the face remained on the screen until the participants chose an answer. Individual response times were not recorded, but the average time required for the encoding tasks was between 8–10 minutes (approximately 3 min/task). Table 1 shows the emotion recognition rates (proportion of correct responses), which were high and ranged from 0.79 to 0.99. This means that accuracy was around 4.7 to 5.9 times higher than the proportion expected by chance guessing (the chance recognition rate in a 6-alternative forced-choice task is 0.16).

**Table 1:** Means (and standard deviations) for emotion recognition rates (proportion of correct responses) in the encoding task.

Modality	Anger	Disgust	Fear	Happiness	Neutral	Sadness	Total
Faces	0.95(0.12)	0.99(0.056)	0.93(0.15)	0.99(0.062)	0.83(0.22)	0.96(0.11)	0.94(0.06)
Voices	0.96(0.10)	0.94(0.12)	0.90(0.16)	0.89(0.18)	0.79(0.25)	0.93(0.14)	0.90(0.08)
Face-voice	0.99(0.05)	0.99(0.01)	0.99(0.05)	0.99(0.05)	0.91(0.17)	0.95(0.11)	0.97(0.04)
**Total**	0.97(0.06)	0.98(0.04)	0.94(0.09)	0.96(0.07)	0.84(0.16)	0.94(0.08)	0.94(0.05)

#### Recognition memory test

The three encoding tasks were directly followed by an equal number of surprise recognition memory tests, in which the participants were presented with the 24 old items from the encoding task interspersed with 24 new stimuli (the identities of which had not been previously encountered in the encoding tasks). The recognition tests were presented in the same fixed order as the encoding tasks (i.e., the face-only condition first, followed by the voice-only and the combined face-voice conditions), and stimuli were presented in random order across subjects within each block. The procedure was similar for all modalities and made use of the Remember/Know paradigm [26].

For each item, the participants were instructed to answer the following question with one of the response options “yes, remember”, “yes, know”, or “no”: “*Have you seen this face before*?” (for faces), “*Have you heard this voice before*?” (for voices), and “*Has this person been present earlier in the experiment*?” (for face-voice combinations). The following instructions were given prior to the memory task: “You will now be presented with a number of faces/voices/face-voice combinations. Some of these have been presented in the earlier task, whereas others are new. Try to remember if you have seen/heard the faces/voices before. If the face/voice/person induces a memory of something that you experienced (e.g., associations, thought, or feelings) at the time you first saw/heard the face/voice/person you should use the response ‘*yes*, *remember’*. If you instead think that the face/voice/person feels familiar, but you cannot remember any details from the last time you saw/heard the face/voice/person, then you should use the response ‘*yes*, *know*’. Finally, if you think that you have not seen/heard the face/voice/person before, you should use the response ‘*no*’.”

Participants made their judgments by clicking the appropriate box on the computer screen using a mouse. Similar to the encoding tasks, there were no time limits and participants were allowed to listen to the voice stimuli as many times as required to reach a decision. Individual response times were not recorded, but the average time required for the three memory tests was between 10–12 minutes (3.5 min/task).

## Results

### Recognition accuracy

We used the discrimination index (*P*_r_) as our measure of recognition accuracy, following Snodgrass and Corvin [38]. *P*_r_ was calculated as the overall hit rate (collapsed across both remember and know responses) minus the false alarm rate.

To test for own-sex bias, we investigated the effects of item sex and participant sex on memory accuracy. Three separate mixed ANOVAs with participant sex as a between-groups variable, and item sex and expression (anger, disgust, fear, happiness, neutral, and sadness) as repeated measures, were conducted for the *P*_r_ rates for faces, voices, and face-voice-combinations. Main effects of expression were significant for all presentation modalities, but the main effect of participant sex was not significant for any modality, which suggests that male and female participants overall performed on a similar level on the memory tasks, and no 3-way interactions were significant. Table 2 shows how overall *P*_r_ rates (for each presentation modality) vary as function of expression for faces, voices, and face-voice combinations, which illustrates the main effects of emotion expression. For the sake of completeness, *P*_r_ rates for each condition (expression, item sex, and participant sex) are displayed in Table 3 separately for each presentation modality.

**Table 2:** Means (and standard deviations) for overall accuracy (*P*_*r*_), remember hits (R), and know hits (K) for each emotion expression and presentation modality.

Modality	Index	Anger	Disgust	Fear	Happiness	Neutral	Sadness
Faces	*P*_r_	0.66(0.26)	0.52(0.31)	0.68(0.27)	0.66(0.28)	0.73(0.26)***	0.56(0.28)
Voices	*P*_r_	0.27(0.31)	0.28(0.29)	0.31(0.32)	0.43(0.33)	0.53(0.31)***	0.30(0.30)
Face-Voice	*P*_r_	0.22(0.33)	0.38(0.32)	0.48(0.32)	0.36(0.33)	0.54(0.28)*	0.42(0.31)
Faces	R	0.55(0.30)	0.50(0.31)	0.55(0.31)	0.52(0.33)	0.61(0.33)***	0.43(0.29)
Voices	R	0.53(0.32)***	0.45(0.28)	0.48(0.29)	0.43(0.28)	0.38(0.31)	0.38(0.27)
Face-Voice	R	0.34(0.28)	0.32(0.28)	0.44(0.29)***	0.38(0.29)	0.34(0.28)	0.31(0.28)
Faces	K	0.28(0.26)	0.28(0.27)	0.30(0.27)	0.26(0.27)	0.25(0.29)	0.32(0.26)**
Voices	K	0.27(0.27)	0.28(0.25)	0.27(0.25)	0.30(0.25)	0.36(0.29)***	0.30(0.25)
Face-Voice	K	0.26(0.24)	0.31(0.26)	0.28(0.26)	0.26(0.23)	0.27(0.24)	0.32(0.27)*

*Notes*. Bold type indicates a value that was significantly different (Bonferroni tests) from the other values in the same row.

*** *p* < .001

** *p* < .01

* *p* < .05

**Table 3:** Means (and standard deviations) for overall accuracy (*P*_*r*_), remember hits (R), and know hits (K) presented separately for each condition (item sex, participant sex, and emotion expression) for each presentation modality.

Modality	Index	Participant and Item Sex	Anger	Disgust	Fear	Happiness	Neutral	Sadness
Faces	*P*_r_	MM	0.65(0.35)	0.47(0.41)	0.62(0.38)	0.66(0.37)	0.69(0.38)	0.57(0.37)
		MF	0.66(0.39)	0.58(0.40)	0.73(0.31)	0.66(0.37)	0.78(0.30)	0.54(0.40)
		FM	0.61(0.37)	0.42(0.44)	0.63(0.39)	0.66(0.37)	0.67(0.35)	0.53(0.38)
		FF	0.70(0.34)	0.61(0.39)	0.73(0.34)	0.64(0.37)	0.78(0.33)	0.59(0.39)
Voices	*P*_r_	MM	0.31(0.46)	0.18(0.39)	0.21(0.46)	0.47(0.41)	0.48(0.40)	0.38(0.41)
		MF	0.26(0.40)	0.46(0.42)	0.41(0.44)	0.38(0.45)	0.57(0.43)	0.17(0.44)
		FM	0.30(0.43)	0.07(0.41)	0.22(0.42)	0.46(0.41)	0.49(0.40)	0.46(0.39)
		FF	0.23(0.44)	0.45(0.42)	0.39(0.46)	0.40(0.47)	0.58(0.43)	0.19(0.42)
Faces-Voice	*P*_r_	MM	0.25(0.45)	0.39(0.42)	0.48(0.44)	0.39(0.44)	0.50(0.40)	0.35(0.43)
		MF	0.24(0.49)	0.35(0.43)	0.47(0.45)	0.32(0.47)	0.58(0.36)	0.43(0.40)
		FM	0.17(0.44)	0.45(0.41)	0.47(0.42)	0.35(0.43)	0.46(0.40)	0.37(0.45)
		FF	0.25(0.46)	0.34(0.46)	0.48(0.40)	0.38(0.46)	0.61(0.37)	0.52(0.40)
Faces	R	MM	0.51(0.38)	0.50(0.39)	0.46(0.39)	0.51(0.40)	0.61(0.40)	0.39(0.37)
		MF	0.63(0.39)	0.52(0.40)	0.62(0.36)	0.50(0.39)	0.60(0.39)	0.44(0.39)
		FM	0.45(0.37)	0.46(0.38)	0.48(0.39)	0.54(0.38)	0.60(0.39)	0.38(0.36)
		FF	0.64(0.36)	0.54(0.39)	0.63(0.36)	0.51(0.39)	0.64(0.38)	0.49(0.36)
Voices	R	MM	0.50(0.37)	0.42(0.35)	0.41(0.36)	0.45(0.35)	0.39(0.37)	0.37(0.36)
		MF	0.59(0.38)	0.49(0.38)	0.50(0.35)	0.42(0.37)	0.43(0.39)	0.38(0.33)
		FM	0.46(0.40)	0.39(0.34)	0.45(0.37)	0.41(0.35)	0.35(0.35)	0.38(0.37)
		FF	0.59(0.39)	0.48(0.36)	0.53(0.38)	0.43(0.37)	0.38(0.38)	0.37(0.33)
Faces-Voice	R	MM	0.35(0.37)	0.34(0.37)	0.43(0.39)	0.37(0.38)	0.35(0.37)	0.31(0.35)
		MF	0.39(0.35)	0.31(0.35)	0.43(0.38)	0.41(0.38)	0.35(0.36)	0.31(0.35)
		FM	0.27(0.34)	0.36(0.38)	0.41(0.36)	0.32(0.36)	0.29(0.35)	0.28(0.32)
		FF	0.37(0.35)	0.26(0.34)	0.47(0.38)	0.44(0.37)	0.37(0.35)	0.33(0.35)
Faces	K	MM	0.27(0.34)	0.25(0.34)	0.36(0.36)	0.25(0.36)	0.26(0.37)	0.31(0.34)
		MF	0.27(0.36)	0.26(0.34)	0.27(0.34)	0.29(0.34)	0.25(0.34)	0.32(0.34)
		FM	0.30(0.33)	0.30(0.35)	0.33(0.35)	0.24(0.31)	0.28(0.36)	0.33(0.34)
		FF	0.27(0.33)	0.30(0.34)	0.26(0.34)	0.27(0.32)	0.22(0.33)	0.30(0.33)
Voices	K	MM	0.26(0.34)	0.26(0.31)	0.26(0.33)	0.27(0.31)	0.32(0.37)	0.26(0.32)
		MF	0.26(0.34)	0.28(0.36)	0.27(0.33)	0.28(0.32)	0.32(0.36)	0.27(0.31)
		FM	0.31(0.34)	0.28(0.32)	0.29(0.33)	0.34(0.34)	0.36(0.36)	0.33(0.35)
		FF	0.26(0.34)	0.28(0.32)	0.26(0.33)	0.29(0.33)	0.40(0.37)	0.30(0.31)
Faces-Voice	K	MM	0.26(0.32)	0.30(0.32)	0.31(0.36)	0.24(0.30)	0.25(0.33)	0.28(0.35)
		MF	0.28(0.34)	0.28(0.35)	0.28(0.33)	0.27(0.34)	0.27(0.34)	0.28(0.33)
		FM	0.27(0.33)	0.35(0.35)	0.32(0.33)	0.25(0.31)	0.29(0.32)	0.35(0.35)
		FF	0.25(0.32)	0.30(0.33)	0.24(0.31)	0.29(0.31)	0.28(0.32)	0.33(0.35)

*Notes*. Male participants, Male items (MM); Male participants, Female items (MF); Female participants, Male items (FM); Female participants, Female items (FF).

**Fig 1:**
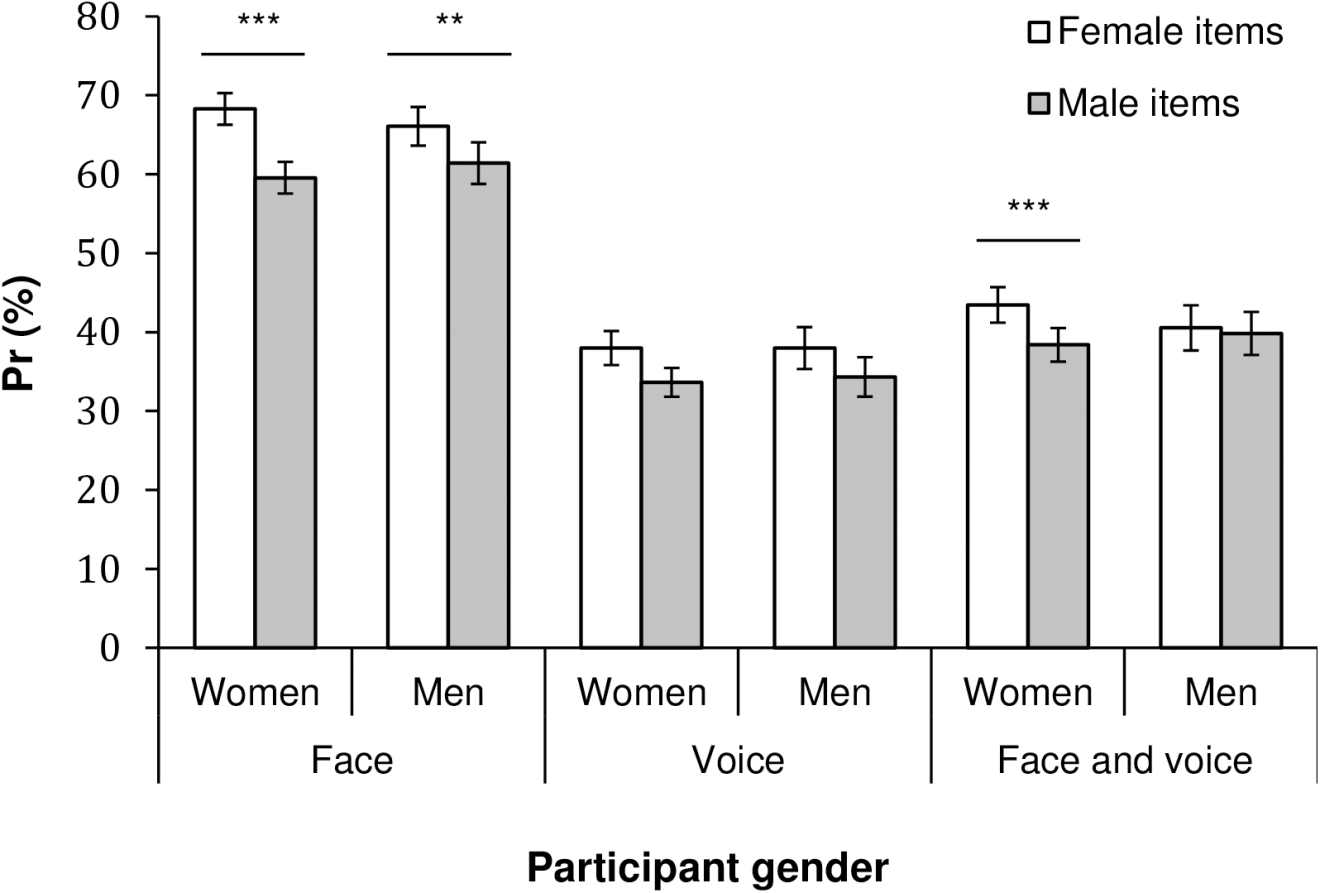
Memory accuracy (*P*_*r*_) as a function of item sex and participant sex for faces, voices and face-voice combinations. The *p*-values indicate post-hoc pairwise comparisons (Bonferroni tests) comparing male and female participants’ accuracy for male and female items, respectively. Error bars represent 95% *CI*. ** *p* = .003, *** *p* < .001.

For faces, we observed a significant main effect of expression, *F*(5, 2960) = 53.40, *p* < .001, η_p_^2^ = .08. This main effect was further explored using post-hoc multiple comparisons (Bonferroni tests) to control for familywise error rates. Recognition was most accurate for neutral faces (*P*_r_ = .73), which were better recognized than fearful faces (.68), which in turn were better recognized than happy and angry faces (.66), which were better recognized than sad (.56) and disgust (.52) faces (Bonferroni tests, *ps* ≤ .002; for post-hoc tests, we report *p*-values corrected for all pairwise combinations throughout the paper). There was also a main effect of item sex, *F*(1, 592) = 61.43, *p* < .001, η_p_^2^ = .09, demonstrating that female faces (*P*_r_ = .67) were more accurately recognized than male faces (.61). The item sex by expression interaction was also significant, *F*(5, 2960) = 8.54, *p* < .001, η_p_^2^ = .01, and post-hoc Bonferroni tests revealed that female faces were better recognized than male faces for disgust, fear, and neutral expressions (*ps* < .001). The effect of main interest was the participant sex by item sex interaction, *F*(1, 592) = 5.25, *p* = .022, η_p_^2^ = .01, which revealed that female participants displayed a memory bias for female faces (Bonferroni tests, *p*s < .003; see Fig 1). Both men and women performed significantly better for female compared to male faces but the difference between accuracy for female and male items was larger for female participants (see Fig 1).

For voices, we also observed a significant main effect of expression *F*(5, 2960) = 64.61, *p* < .001, η_p_^2^ = .10. Neutral voices (*P*_r_ = .53) were better recognized than all emotional voices (Bonferroni tests, *p*s < .001), followed by happy voices (.43) which in turn were better recognized (*p*s < .001) than the remaining voices. A significant main effect of item sex, *F*(1, 592) = 14.94, *p* < .001, η_p_^2^ = .02, showed that female voices (*P*_r_ = .38) were better recognized than male voices (.34). The item sex by expression interaction was also significant *F*(5, 2960) = 68.57, *p* < .001, η_p_^2^ = .10, and post-hoc Bonferroni tests revealed that female voices were better recognized than male voices for disgust, fear, and neutral expressions (*p*s < .013), but sad male voices were better recognized than sad female voices (*p* < .001). With regard to own-sex bias, the participant sex by item sex interaction was not significant for voices, *F*(1, 592) = 0.18, *p* = .672. As shown in Fig 1, both female and male participants overall performed better for female vs. male items.

Finally, for the face-voice combinations, the main effect of expression was significant *F*(5, 2960) = 73.81, *p* < .001, η_p_^2^ = .11, where neutral stimuli (*P*_r_ = .54) were better recognized than all other expressions (Bonferroni tests, *p*s ≤. 005), followed by fearful, sad, disgusted and happy stimuli which in turn were better recognized than angry stimuli (*ps* < .001). The main effect of item sex was also significant, *F*(1, 592) = 7.49, *p* = .006, η_p_^2^ = .01, and female items (*P*_r_ = .42) were better recognized than male items (.39). The item sex by expression interaction, *F*(5, 2960) = 8.54, *p* < .001, η_p_^2^ = .01, showed that neutral and sad female face-voice combinations were better recognized than male ones (Bonferroni tests, *ps* < .001). The participant sex by item sex interaction was also significant, *F* (1, 592) = 5.51, *p* = .019, η_p_^2^ = .01, and again revealed own-sex bias for female participants. As shown in Fig 1, only female participants displayed significantly better accuracy for female compared to male face-voice combinations (Bonferroni test, *p* < .001).

### Remember/Know responses

We tested for own-sex bias in participants’ subjective feelings of recollection (remember hits) and familiarity (know hits) in a similar way as we did for memory accuracy. Separate mixed ANOVAs were conducted, with participant sex as a between-groups variable and item sex and expression (6 levels) as repeated measures factors, for remember and know rates for faces, voices and face-voice-combinations. Main effects of expression were significant for both remember and know responses in all presentation modalities, the main effect of participant sex was significant only for know responses in the vocal modality (detailed below), and no 3-way interactions were significant. Hit rates were not corrected for false alarms in these analyses because we were primarily interested in the participants’ subjective feelings of recollection and familiarity, respectively. Remember and know rates for each presentation modality are shown in Table 2 (as a function of emotion expression) and Table 3 (as a function of expression, item sex, and participant sex).

#### Remember responses

**Fig 2:**
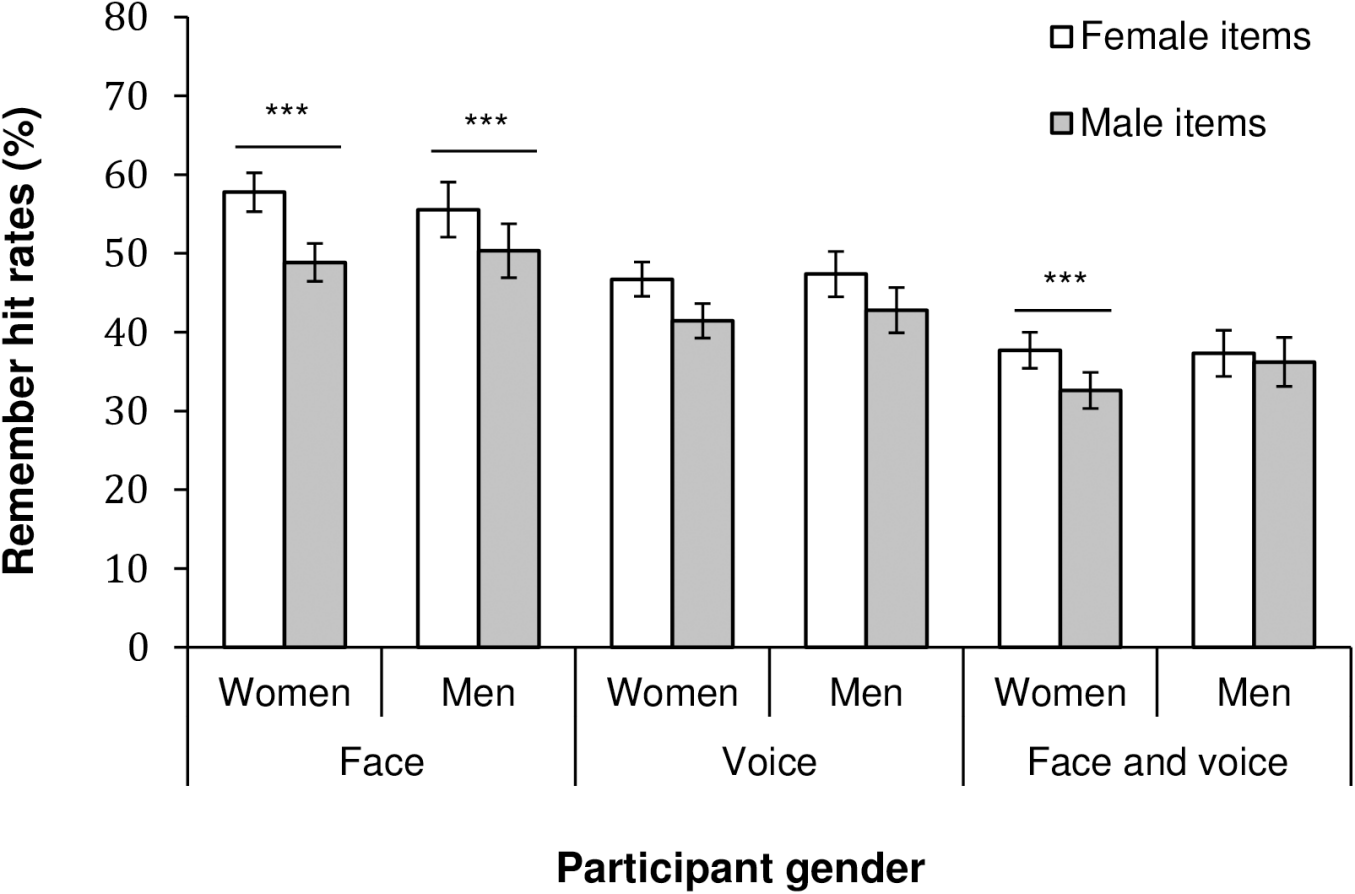
Recollection hit rates as a function of item sex and participant sex for faces, voices and face-voice combinations. The *p*-values indicate post-hoc pairwise comparisons (Bonferroni tests) comparing male and female participants’ recollection rates for male and female items, respectively. Error bars represent 95% *CI*. *** *p* < .001.

For remember hits of faces, we observed significant main effects of both expression *F*(5, 2960) = 39.31, *p*< .001, η_p_^2^ = .06, and item sex, *F*(1, 592) = 77.61, *p* < .001, η_p_^2^ = .12. For the main effect of expression, post-hoc multiple comparisons (Bonferroni tests) revealed that neutral faces (remember hit rate, *M* = .62) evoked significantly more remember hits than did angry (.56), fearful (.55), happy (.52), and disgusted faces (.51), which in turn had higher recollection rates than sad faces (.43, all *ps* ≤ .001). The main effect of item sex suggests higher recollection rates for female (*M* = .57) compared to male faces (.50). The item sex by expression interaction was significant *F*(5, 2960) = 14.53, *p* < .001, η_p_^2^ = .02, and post-hoc analyses showed that female faces had significantly higher recollection rates than male faces for anger, fear, and sadness (Bonferroni tests, *ps* < .001). The participant sex by item sex interaction effect was also significant, *F*(1, 592) = 5.25, *p* = .022, η_p_^2^ = .01, and demonstrates an own-sex bias for female participants as illustrated in Fig 2. Both male and female participants showed higher remember rates for female vs. male items, but the difference between female and male items was larger for female participants (Bonferroni test, *p* < .001).

For the recollection of voices, the main effect of expression was significant *F*(5, 2960) = 33.27, *p* < .001, η_p_^2^ = .05. Post-hoc Bonferroni tests (all *ps* < .001) indicated that recollection of angry voices (remember hit rate, *M* = .54) was higher than for all other expressions, and that fearful (.48) and disgusted (.46) voices evoked significantly more remember hits than did happy (.44), neutral (.39) or sad (.38) vocalizations. The main effect of item sex was significant, *F*(1, 592) = 41.64, *p* < .001, η_p_^2^ = .07, demonstrating that the remember rates for female voices (*M* = .47) were higher than the rates for male voices (.42). The item sex by expression interaction *F*(5, 2960) = 6.71, *p* < .001, η_p_^2^ = .01, revealed that angry, disgusted and fearful female voices were better recollected than the corresponding male voices (Bonferroni tests, *ps* ≤ .001). However, the participant sex by item sex interaction was not significant here, *F*(1, 592) = 0.22, *p* = .640. Overall, both female and male participants showed higher rates for female items than for male items, see Fig 2.

For face-voice combinations, there was a significant main effect of expression *F*(5, 2960) = 24.31, *p* < .001, η_p_^2^ = .04, which showed that fearful expressions (remember hit rate, *M* = .44) received higher recollection rates than all other expressions, followed by happy expressions (.39) which in turn received higher rates than all remaining expressions except anger (.35; Bonferroni tests, *ps ≤* .004). The main effect of item sex was also significant, *F*(1, 592) = 14.51, *p* < .001, η_p_^2^ = .02, where female items (*M* = .38) were better recollected than male items (.35). The item sex by expression interaction was significant, *F*(5, 2960) = 7.70, *p* < .001. η_p_^2^ = .01. Post-hoc Bonferroni tests demonstrated that angry and happy female face-voice combinations showed higher recollection rates versus male items (*p*s < .001), whereas for disgust, recollection of male items was higher than for female items (*p =* .005). Here the participant sex by item sex interaction was also significant, *F*(1, 592) = 6.06, *p* = .014, η_p_^2^ = .01, and demonstrated that female, but not male, participants displayed a memory bias for female face-voice combinations (Bonferroni test, *p* < .001; see Fig 2).

#### Know responses

**Fig 3:**
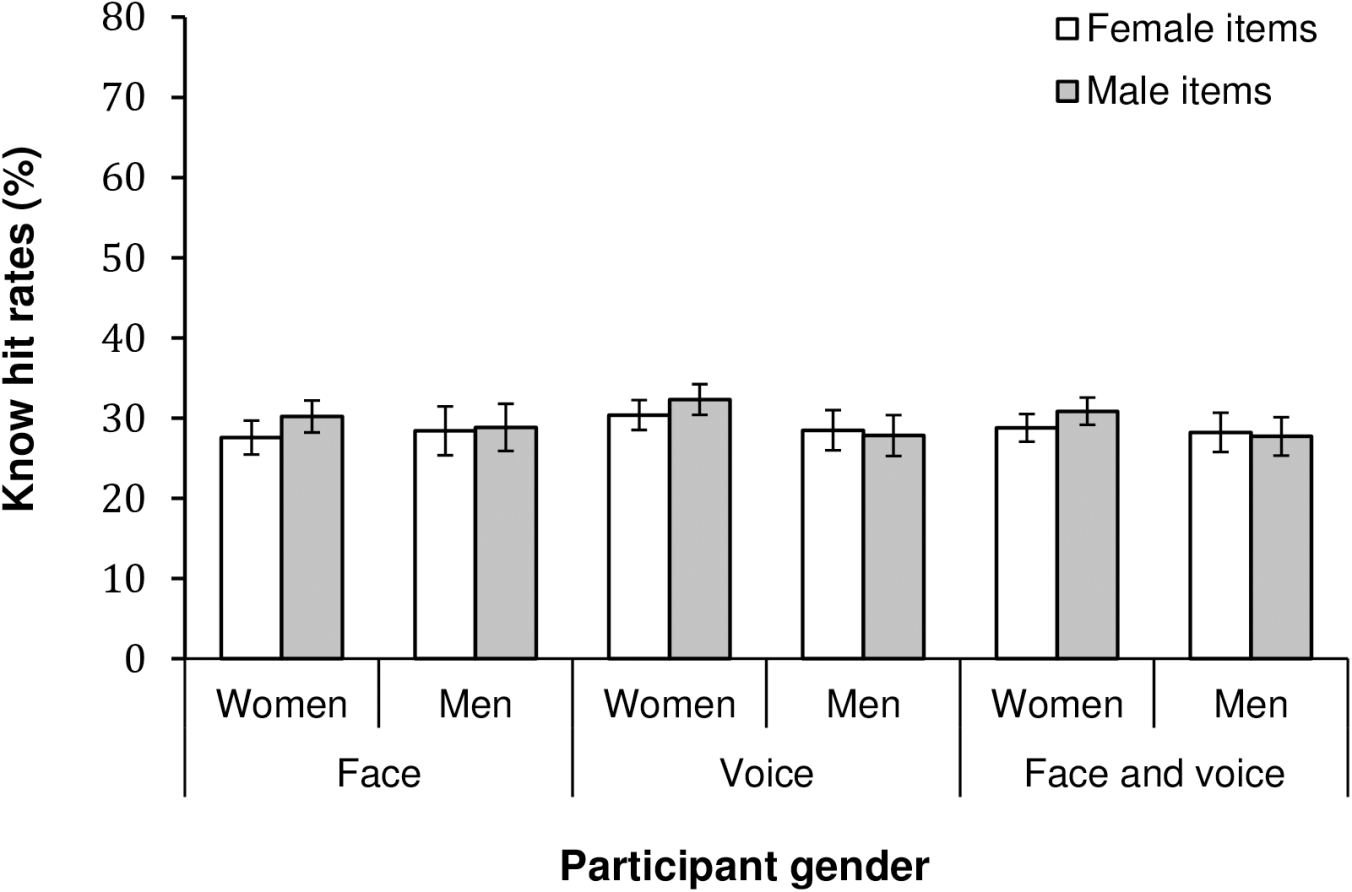
Familiarity hit rates as a function of item sex and participant sex for faces, voices and face-voice combinations. Error bars represent 95% *CI*.

Regarding know responses for faces, we observed a significant main effect of expression, *F*(5, 2960) = 7.03, *p* < .001, η_p_^2^ = .01. Sad expressions received the highest familiarity rates (know hit rate, *M* = .32), followed by fearful (.31), disgusted and angry (.29), happy (.27) and neutral (.26) faces. Post-hoc Bonferroni tests showed that sad faces had higher familiarity rates than happy and neutral expressions (*ps* ≤ .002). There was a main effect of item sex *F*(1, 592) = 4.19, *p* = .041, η_p_^2^ = .01, revealing that male faces (*M* = .30) were overall more familiar than female faces (.28). The item sex by expression interaction was also significant, *F*(5, 2960) = 4.39, *p* < .001 (Greenhouse-Geisser corrected), η_p_^2^ = .01, and showed that male fearful items were more familiar than female items (*p*s < .001, Bonferroni tests). The participant sex by item sex interaction was not significant, *F*(1, 592) = 2.13, *p* = .145 (see Fig 3).

For voices, the main effect of expression was significant *F*(5, 2960) = 9.33, *p* < .001, η_p_^2^ = .02. Post-hoc Bonferroni tests (*ps* ≤ .001) revealed that neutral voices (know hit rate, *M* = .36) had higher familiarity rates than all the emotional voices. There was also a significant main effect of participant sex *F*(1, 592) = 5.42, *p* = .020, η_p_^2^ = .01, which demonstrated that female participants (*M* = .32) performed significantly better than male participants (.28) according to familiarity rates. The participant sex by item sex interaction effect was not significant, *F*(1, 592) = 2.72, *p* = .100, see Fig 3.

Finally, for know responses of face-voice combinations, we observed a main effect of expression, *F*(5, 2960) = 5.50, *p* < .001, η_p_^2^ = .01. Post-hoc Bonferroni tests (*p*s ≤ .02) demonstrated that the familiarity of sad face-voice combinations (know hit rate, *M* = .32) was statistically higher than for neutral (.28), and happy and angry (.27) expressions. We also observed significant item sex by expression, *F*(5, 2960) = 2.84, *p* = .014. η_p_^2^ = .01, and participant sex by expression, *F*(5, 2960) = 2.25, *p* = .047 (Greenhouse-Geisser corrected), η_p_^2^ = .004, interaction effects (for values, see Table 3). However, for these effects post-hoc Bonferroni tests revealed no significant pairwise differences. The participant sex by item sex interaction effect was not significant *F*(1, 592) = 2.89, *p* = .090, as seen in Fig 3.

### Additional analyses

Because the emotion recognition rates in the encoding tasks showed some variability across conditions, we calculated the correlation between the emotion recognition rate for each item that was included in the encoding task and its corresponding *P*_r_ values. This correlation was not significant, *r* = -.10, *p* = .410 (*N* = 72), which suggests that memory performance was not associated with the ease or difficulty of emotion recognition in our sample.

## Discussion

This study aimed to address three interrelated research questions about the effects of sex and emotion expression on memory for faces and voices, presented alone and in combination. The first aim was to investigate own-sex bias. As predicted, results showed evidence for own-sex bias, to the effect that female participants performed relatively better for female faces compared to male participants. We also observed female own-sex bias for face-voice combinations, whereas for voices both men and women recognized female items with higher accuracy than male items. The second aim was to investigate the effects of emotion expression on recognition memory. Here, results were similar for faces, voices, and face-voice combinations, and showed that neutral expressions had higher accuracy rates compared to emotional expressions. The accuracy for specific emotions, however, differed across presentation modalities. Finally, the third aim was to use the Remember/Know paradigm to investigate how the subjective sense of recollection and familiarity varies as a function of sex and emotion expression. In contrast to the findings for memory accuracy, we observed the highest recollection rates for emotional (anger and fear) items for voices and face-voice combinations, although neutral items received the highest recollection rates for faces. This indicates that emotions may enhance the subjective sense of recollection without enhancing the accuracy of recognition memory. Finally, own-sex bias was observed for recollection rates but not for familiarity rates, which suggests that own-sex bias may depend mainly on recollection processes.

### Sex effects in memory for emotional faces and voices

We did not observe any overall memory advantage for female participants over male participants, contrary to several studies reviewed in [2]. It has been suggested that women may implicitly allocate more attention to the expression of faces than men [12], which entails more focus on local facial features relevant to emotional expressions such as the mouth and the eyes [39], whereas men may process faces more holistically or globally. This reasoning has received some support from data showing that face recognition in men improved when they were explicitly instructed to attend to the expression of faces [12]. In the current study, the encoding task consisted of an emotion recognition task, and we speculate that this task could have increased focus on expression features for men and women alike, which could have enhanced men’s overall performance compared to the studies reviewed in [2].

It has been suggested that female neutral faces are easier to recall in part because they are more distinctive than male faces [3]. In the current study, both men and women overall recognized female stimuli better than male stimuli, although this advantage was not as pronounced as previous studies have reported (e.g., [3, 10]). We observed recognition advantage for female neutral items for all presentation modalities, and also for several emotion categories (i.e., disgust and fear for faces and voices, and sadness for face-voice combinations). It thus appears that although the recognition advantage for female items was present also for emotional items, it was less consistent for emotional compared to neutral items. A possible explanation for this observation could be that emotional expressions make female and male items more similar in terms of their distinctiveness, but more research is needed to understand the mechanisms behind memory advantage for female items.

We analyzed the interaction between item sex and participant sex, replicating previous findings of own-sex bias for female participants, who displayed relatively higher memory accuracy for female vs. male faces, in comparison to male participants (e.g., [2, 10]). Although the underlying causes for this effect are not fully known, it has been suggested that women may attend and react more to female faces due to complex interactions of social and biological mechanisms [2], resulting in higher efficiency in encoding and retrieval processes [7, 10, 11]. Regarding biological mechanisms, Lovén et al. [13] demonstrated that activation of fusiform gyrus–which is associated with perception of faces [40]–is associated with female own-sex bias during encoding of neutral faces. Furthermore, amygdala activation in socioemotional memory may differ for men and women. For women, the left amygdala is more activated during the encoding of female faces, compared to male faces, but for men, the right amygdala is more activated during the encoding of male faces [14]. Our results also demonstrated, for the first time, female own-sex bias in memory for face-voice combinations, but no own-sex bias was apparent for voices. It thus remains a possibility that the own-sex bias for face-voice combinations was driven mainly by the facial component. We note that it has been suggested that information about sex and identity appear to be processed independently for faces, whereas this may not be the case for voices [41]. How (and why) memory biases for faces and voices differ remains an exciting topic for future research.

Turning next to the subjective sense of recollection, remember responses indicated a female own-sex bias for both faces and face-voice combinations, but there was no memory bias for know responses. Our findings thus suggest that it is recollection processes–rather than familiarity processes–that play a key role in own-sex bias. Notably, this pattern has also been found in studies of own-group biases in face recognition [42], which suggests that the mechanisms behind own-sex biases may be more similar to the mechanisms of own-race biases than previously thought. According to Palmer et al. [7], women’s recognition of female faces relies on attention at encoding, and divided attention manipulations at this phase decreases women’s recollection ratings of female faces more than for male faces. In our study, there were no attention manipulation tasks, which facilitated recollection rates, and this may explain why we found a memory bias only for recollection, and not familiarity, rates. Importantly, our findings contribute to the growing evidence showing a robust female own-sex bias even when manipulations vary between studies [2].

Finally, our results suggested that female own-sex bias can be observed for neutral as well as emotional items. However, in the absence of item sex × participant sex × expression interactions, the pattern of results do not give any clear indications about how own-sex bias varies as a function of emotion. Effects of emotional expressions on memory are discussed in detail below.

### Effects of emotion expression on memory for faces and voices

While emotional expressions are generally thought to facilitate social memory [18], our findings suggest this may not always be the case. Using a novel design where several emotion categories and neutral stimuli were included in the same test, we instead observed higher memory accuracy for neutral stimuli than for emotional stimuli. Our results are similar to what Johansson et al. [23] previously observed for faces, but we extend this observation to both faces and voices, and face-voice combinations as well. This suggests that the memory advantage for neutral stimuli was not specific to any presentation modality. This finding underscores the need for further research about the conditions in which neutral and emotional expressions, respectively, facilitate memory for socioemotional information. We utilized an emotion recognition task at encoding, and we speculate that this type of task may have preferentially focused participants’ attention on emotional features, leading to less attention on the identity features of emotional stimuli. Neutral stimuli may also have attracted attention because they stood out because of their relative novelty, given that emotional stimuli outnumbered neutral stimuli by five to one in our task. We also note that neutral expressions received slightly lower emotion recognition rates in the encoding task compared to the emotional expressions. However, we found no correlation between emotion recognition accuracy and memory performance, which suggests that it is unlikely that differences in emotion recognition rates would underlie differences in memory accuracy.

We further documented higher memory accuracy for some emotions than for others, suggesting that the processing of emotional stimuli varied across emotion categories. In particular, memory accuracy was in general higher for happiness and fear stimuli than for the other non-neutral expressions–although we note that accuracy for specific emotions also varied across presentation modalities. We speculate that the relatively high accuracy rates for happy expressions may be due to attentional biases toward pro-social stimuli [20], which signal approachability and approval [17]. Fear expressions are instead associated with threat to one’s well-being and survival, and may attract prioritized processing resources due to their adaptive significance [22].

Interestingly, the pattern of results for the subjective sense of recollection differed from the pattern for memory accuracy rates. We observed that fearful and angry stimuli received the highest recollection rates for voices and face-voice combinations, although for faces neutral stimuli again had the highest recollection rate. In this sense, anger stimuli stood out with high rates of remember responses, but low memory accuracy–which indicates that angry items received a relatively high degree of false positives in the recognition memory task (especially for voices and face-voice combinations). It can be speculated that angry stimuli may have received relatively less attention during encoding because they signal disapproval and social threat. However, during the memory task, angry items may instead have preferentially increased participants’ arousal, thereby increasing their likelihood to choose remember responses. Regarding the subjective sense of familiarity (know rates), sad stimuli had among the lowest recollection rates, yet the highest familiarity rates. It is possible that sad expressions were perceived as signaling a lower arousal affective state and thus failed to reach conscious recollection, possibly due to a more general assessment between encoding and retrieval of sad items [23]. This is consistent with studies that propose that sadness prompts empathic reactions (e.g., [43]), resulting in recall of the expression rather than the identity. Overall, the patterns of results for recollection and familiarity rates are in line with Phelps and Sharot [44], who have suggested that emotion enhances the subjective feeling of recollection but does not necessarily increase memory accuracy. Neutral stimuli may instead enhance recognition accuracy–for example by evoking a variety of contextual, often perceptual, details–but do not enhance the subjective feeling of remembering to the same extent as emotional stimuli, perhaps because they are not equally associated with arousal [28]. Notably, we expand upon previous findings by showing that the dissociation between objective and subjective remembering can also be observed for stimuli consisting of emotionally expressive faces and voices.

### Similarities and differences between memory for face and voices

Our aim was to compare the pattern of results across the face, voice, and face-voice conditions, and these results have been discussed above. However, we did not aim to directly compare the level of performance in the different presentation modalities, and did not design our study for this purpose. Nevertheless, in accordance with previous research [45], results indicated that faces (*P*_r_ = .64) were more accurately remembered than face-voice combinations (*P*_r_ = .41), which in turn were more accurate than voices (*P*_r_ = .36). The advantage for faces appears strong, but we note that a presentation order effect may have contributed to this finding. We assessed faces first, which may have artificially increased accuracy relative to voices and face-voice combinations due to more fatigue in the latter conditions. The recognition memory tasks may also have been more demanding for the voice and face-voice conditions, compared to the face only condition. For example, the tasks preceding face memory test consisted of the voice emotion and the face-voice-combination emotion recognition test, whereas the tasks prior to the face-voice-combination memory test were the face memory test and the voice memory test. In addition, previous research has reported that voice recognition is more vulnerable to distractor items compared to face recognition (e.g., [33]), and we note that this so called face primacy effect may also have contributed to the relatively higher recognition memory for faces compared to the other presentation modalities. Finally, stimulus presentation times at encoding were not kept constant across conditions in the current study, because participants were allowed to repeat the playback of voice only stimuli as many times as needed to reach a decision. This may have preferentially affected memory for voices, and thus helps to complicate a comparison of performance levels across presentation modalities.

## Limitations

The current study is subject to several limitations. First, we acknowledge that our results are based on a limited stimulus set, with few exemplars for each combination of item sex, emotion expression, and presentation modality. We therefore encourage replication attempts in order to find out how well the pattern of results generalizes to other stimulus sets. Replication using naturalistic and/or dynamic emotion expressions (e.g., short videos of persons expressing emotions through their faces and voices) would be especially worthwhile. A second potential limitation is the fact that we did not measure reaction times in the encoding and memory tasks. Future studies should include such measures in order to investigate, for example, possible sex differences in the time allotted to various emotion expressions at encoding and how such possible differences map onto sex differences in memory performance. This enterprise could lead to important information about the mechanisms underlying the observed effects of sex and emotion expression. For facial expressions, studies could further utilize eye-tracking methods to explore possible effects of sex and emotion on gaze patterns at encoding and subsequent memory performance. A third potential drawback of the present study is that participants did not rate the stimuli according to attractiveness or distinctiveness. Some studies report that unattractive faces are recalled more often than attractive faces and that this relation is mediated by distinctiveness [46, 47]. Thus, it is possible that in our study attractiveness and distinctiveness account, at least partially, for the memory advantage of neutral items and not the depicted expression per se. How attractiveness and distinctiveness ratings of faces and voices vary as a function of emotional expression and item sex remains an interesting topic for future studies on social memory.

## Conclusions

Our results suggest that memory for faces and voices are influenced by the expressions that they convey, with higher accuracy for neutral vs. emotional stimuli, and that emotional displays can increase the sense of recollection without increasing accuracy. In addition, participants overall displayed higher memory accuracy for female stimuli than for male stimuli, with female own-sex bias for both faces and face-voice combinations. Own-sex memory bias possibly relies on recollection rather than familiarity processes. In conclusion, we argue that our findings highlight the importance of jointly considering effects of expressed emotion, presentation modality, and sex in studies of social memory.

## Supporting information

S1 Table Stimulus identifiers for encoding and recognition tasks. (DOCX)
