# Supplementary material for: Memory for faces and voices varies as a function of sex and expressed emotion
Source: PLoS One. 2017 Jun 1;12(6):e0178423. doi: 10.1371/journal.pone.0178423 (PMC5453523; doi:10.1371/journal.pone.0178423)
Supplement: S1 Table — (DOCX) [file pone.0178423.s001.docx]

**S1 Table. Stimulus identifiers for encoding and recognition tasks.**

| **Encoding task** | |  | | |  | |
| --- | --- | --- | --- | --- | --- | --- |
| Faces | | Voices | | | Face-voice | |
| 020_y_f_a_a.jpg  026_m_m_a_a.jpg  089_y_m_a_a.jpg  139_m_f_a_a.jpg  081_y_m_d_a.jpg  122_m_f_d_a.jpg  126_m_m_d_a.jpg  152_y_f_d_a.jpg  062_y_m_f_a.jpg  077_m_m_f_a.jpg  111_m_f_f_a.jpg  177_y_f_f_a.jpg  048_y_f_h_a.jpg  051_m_m_h_a.jpg  052_m_f_h_a.jpg  105_y_m_h_a.jpg  103_m_f_n_a.jpg  109_y_m_n_a.jpg  115_y_f_n_a.jpg  165_m_m_n_a.jpg  054_y_f_s_a.jpg  072_y_m_s_a.jpg  094_m_m_s_a.jpg  156_m_f_s_a.jpg | | AngFAU12.wav  AngFUS1.wav  AngMIN4.wav  AngMUS7.wav  DisgFUS3.wav  DisgFUS13.wav  DisgMUS6.wav  DisgMSI12.wav  FeaFUS11.wav  FeaFUS15.wav  FeaMIN8.wav  FeaMUS17.wav  HapFIN7.wav  HapFKE18.wav  HapMKE12.wav  HapMKE10.wav  NeuFIN7.wav  NeuFIN9.wav  NeuMUS17.wav  NeuMIN2.wav  SadFIN3.wav  SadFUS21.wav  SadMUS10.wav  SadMUS16.wav | | | 162_y_f_a_a.jpg, AngFUS11.wav  147_y_m_a_a.jpg, AngMUS4.wav  043_m_f_a_a.jpg, AngFUS13.wav  116_m_m_a_a.jpg, AngMUS19.wav  022_y_f_d_a.jpg, DisgFIN3.wav  167_y_m_d_a.jpg, DisgMIN8.wav  157_m_f_d_a.jpg, DisgFUS15.wav  104_m_m_d_a.jpg, DisgMUS20.wav  085_y_f_f_a.jpg, FeaFIN9.wav  008_y_m_f_a.jpg, FeaMIN2.wav  128_m_f_f_a.jpg, FeaFUS2.wav  149_m_m_f_a.jpg, FeaMIN4.wav  132_y_f_h_a.jpg, HapFAU15.wav  170_y_m_h_a.jpg, HapMIN1.wav  011_m_f_h_a.jpg, HapFSI10.wav  136_m_m_h_a.jpg, HapMSI2.wav  063_y_f_n_a.jpg, NeuFIN3.wav  041_y_m_n_a.jpg, NeuMUS4.wav  117_m_f_n_a.jpg, NeuFAU12.wav  058_m_m_n_a.jpg, NeuMAU18.wav  090_y_f_s_a.jpg, SadFIN7.wav  175_y_m_s_a.jpg, SadMIN2.wav  113_m_f_s_a.jpg, SadFUS5.wav  045_m_m_s_a.jpg, SadMUS4.wav | |
| **Recognition task (new stimuli)** | | |  | | |  |
| Faces | Voices | | | Face-voice | | |
| 098_y_f_a_a.jpg  013_y_m_a_a.jpg  006_m_f_a_a.jpg  007_m_m_a_a.jpg  173_y_f_d_a.jpg  153_y_m_d_a.jpg  138_m_f_d_a.jpg  179_m_m_d_a.jpg  134_y_f_f_a.jpg  123_y_m_f_a.jpg  050_m_f_f_a.jpg  056_m_m_f_a.jpg  150_y_f_h_a.jpg  127_y_m_h_a.jpg  064_m_f_h_a.jpg  082_m_m_h_a.jpg  071_y_f_n_a.jpg  037_y_m_n_a.jpg  180_m_f_n_a.jpg  038_m_m_n_a.jpg  171_y_f_s_a.jpg  049_y_m_s_a.jpg  019_m_f_s_a.jpg  070_m_m_s_a.jpg | AngFAU14.wav  AngFAU15.wav  AngMUS6.wav  AngMAU20.wav  DisgFAU12.wav  DisgFAU17.wav  DisgMUS19.wav  DisgMUS9.wav  FeaFIN7.wav  FeaFUS3.wav  FeaMUS9.wav  FeaMAU18.wav  HapFUS2.wav  HapFAU17.wav  HapMKE8.wav  HapMKE6.wav  NeuFUS11.wav  NeuFUS12.wav  NeuMUS16.wav  NeuMUS10.wav  SadFIN10.wav  SadFUS11.wav  SadMAU20.wav  SadMUS19.wav | | | 182_y_f_a_a.jpg, AngFUS3.wav  114_y_m_a_a.jpg, AngMUS20.wav  093_m_f_a_a.jpg, AngFIN3.wav  155_m_m_a_a.jpg, AngMSI9.wav  010_y_f_d_a.jpg, DisgFUS2.wav  031_y_m_d_a.jpg, DisgMAU18.wav  061_m_f_d_a.jpg, DisgFUS21.wav  087_m_m_d_a.jpg, DisgMIN1.wav  069_y_f_f_a.jpg, FeaFIN3.wav  144_y_m_f_a.jpg, FeaMAU10.wav  029_m_f_f_a.jpg, FeaFAU12.wav  159_m_m_f_a.jpg, FeaMUS16.wav  140_y_f_h_a.jpg, HapFIN3.wav  025_y_m_h_a.jpg, HapMIN8.wav  168_m_f_h_a.jpg, HapFUS11.wav  032_m_m_h_a.jpg, HapMUS17.wav  163_y_f_n_a.jpg, NeuFAU17.wav  066_y_m_n_a.jpg, NeuMUS8.wav  035_m_f_n_a.jpg, NeuFUS14.wav  108_m_m_n_a.jpg, NeuMIN4.wav  028_y_f_s_a.jpg, SadFUS3.wav  160_y_m_s_a.jpg, SadMUS6.wav  073_m_f_s_a.jpg, SadFIN5.wav  142_m_m_s_a.jpg, SadMUS20.wav | | |
